# Hormetic dose response to _L_-ascorbic acid as an anti-cancer drug in colorectal cancer cell lines according to SVCT-2 expression

**DOI:** 10.1038/s41598-018-29386-7

**Published:** 2018-07-27

**Authors:** Sungrae Cho, Jin Sung Chae, Hocheol Shin, Yujeong Shin, Haeun Song, Youngwook Kim, Byong Chul Yoo, Kangsan Roh, Seungchan Cho, Eui-joon Kil, Hee-seong Byun, Sang-ho Cho, Seyeon Park, Sukchan Lee, Chang-Hwan Yeom

**Affiliations:** 1 Department of Genetic Engineering, Sungkyunkwan University, Suwon, 16419 Republic of Korea; 2 Yeom Chang-Hwan hospital, Seoul, 06605 Republic of Korea; 3 Department of Applied Chemistry, Dongduk Women’s University, Seoul, 02748 Republic of Korea; 4 Department of Health Sciences and Technology, Samsung Advanced Institute for Health Sciences and Technology, Sungkyunkwan University, Seoul, 06351 Republic of Korea; 5 Colorectal Cancer Branch, Division of Translational and Clinical Research, Research Institute, National Cancer Center, Goyang, 10408 Republic of Korea

## Abstract

_L_-Ascorbic acid (vitamin C, AA) exhibits anti-cancer effects with high-dose treatment through the generation of reactive oxygen species (ROS) and selective damage to cancer cells. The anti-cancer effects of _L_-ascorbic acid are determined by sodium-dependent vitamin C transporter 2 (SVCT-2), a transporter of _L_-ascorbic acid. In this study, we demonstrate that _L_-ascorbic acid treatment showed efficient anti-cancer activity in cell lines with high expression levels of SVCT-2 for a gradient concentration of _L_-ascorbic acid from 10 μM −2 mM. However, in low SVCT-2 expressing cell lines, high-dose _L_-ascorbic acid (>1 mM) showed anti-cancer effects but low-dose (<10 μM) treatment induced cell proliferation. Such conflicting results that depend on the concentration are called a hormetic dose response. A hormetic dose response to low-dose _L_-ascorbic acid was also observed in high SVCT-2 expressing cell lines in the presence of a SVCT family inhibitor. Insufficient uptake of _L_-ascorbic acid in low SVCT-2 expressing cancer cell lines cannot generate sufficient ROS to kill cancer cells, resulting in the hormetic response. Molecular analysis confirmed the increased expression of cancer proliferation markers in the hormetic dose response. These results suggest that _L_-ascorbic exhibits a biphasic effect in cancer cells depending on SVCT-2 expression.

## Introduction

_L_-Ascorbic acid (Vitamin C, AA), which is known as an antioxidant, acts as a pro-oxidant in cancer cells and selectively kills cancer cells when administered at a high dose^1^. Through various studies, the anti-cancer effects of _L_-ascorbic acid were shown to be mediated by inhibition of cellular proliferation and growth through the generation of reactive oxygen species (ROS) and hydrogen peroxide-mediated effects on *in vitro* systems^2–6^. ROS induce cellular damage and induce oxidative stress in cancer cells depending on redox status and metabolism^7,8^. In *in vivo* systems, a pharmacologic dose of _L_-ascorbic acid acted as pro-oxidant and showed anti-cancer effects with generation of ascorbate radicals^9,10^. Many researchers have investigated the mechanism of high-dose _L_-ascorbic acid therapy through ROS generation, which affects cytochrome c release in mitochondria and finally leads to apoptosis^7,11^.

Historically, _L_-ascorbic acid was first recognized as a potential cancer therapeutic agent by *Linus Pauling* and *Ewan Cameron* in 1976^12^. High-dose _L_-ascorbic acid therapy increased the average survival time in previous studies^13^. However, a study conducted in the Mayo clinic showed that _L_-ascorbic acid therapy has no benefits in cancer patients^14,15^. As a possible explanation for this discrepancy, a recent study suggested that sodium-dependent vitamin C transporter family 2 (SVCT-2) is an indicator for high-dose _L_-ascorbic acid therapy by regulating the uptake of _L_-ascorbic acid uptake. Moreover, another study suggested that the metabolic state of cancer cells might be linked to the efficacy of high-dose _L_-ascorbic acid therapy^16^. However, various questions still remain regarding previous controversial clinical studies^12–15^, including (1) the sufficient dose of _L_-ascorbic acid for various cancer cells, (2) the reason behind the poor survival rate of patients treated with high-dose _L_-ascorbic acid, which was even lower than that of the placebo group, in the Mayo Clinic study^14,15^; and (3) whether the anti-cancer activity of _L_-ascorbic acid changes when the plasma concentration of the delivered _L_-ascorbic acid decreases and is maintained at a low level for about 4 hours in blood^17^ due to spontaneous oxidization over a short period of time^18^.

We hypothesized that these issues can be explained by the hormetic dose response, which is also known as the biphasic dose response in the pharmacological concept and is explained by a U-shaped curve^19^, that was observed in several cancer research studies^20–26^. To address these questions, we proposed the hypothesis that in a gradient concentration of _L_-ascorbic acid with different expression levels of SVCT-2, the anti-cancer effects of _L_-ascorbic acid change to hormetic proliferation when insufficient ROS are generated. Since insufficient ROS for cellular apoptosis promotes proliferation of cancer cells through insulin-like growth factor-1^27^ and activation of the Ras gene^28^, we propose that insufficient uptake of _L_-ascorbic results in proliferation of cancer cells.

## Materials and Methods

### Cell culture and reagents

Human colorectal cancer cell lines including Sw620, Sw480, HCT15, HCT116, DLD-1, LoVo, CoLo-205 were purchased from ATCC and human colorectal cancer cell line SNU-C4 and SNU-C5 were purchased from Korea Cell Line Bank (Seoul, Korea). Human colorectal cancer cells were cultured in RPMI1640 media (Gilbco, Cergy Pontoise, France) with 10% fetal bovine serum (Pan Biotech, Aidenbach, Germany) and 1% Penstrep (Pan Biotech) at 37 °C in a humidified incubator with 5% CO_2_. _L_-Ascorbic acid was purchased from BCWorld Pharm. Co. (Seoul, Korea) and phloretin was purchased from Sigma Aldrich (St. Louis, MO, USA).

### Cell viability assay

Cell viability was measured by Neutral Red assay (Sigma). Cells (1 × 10^4^/well) were seeded and cultured in 96-well plates and incubated for 24 hours. Calls were treated with _L_-ascorbic acid for 4 hours, washed with phosphate buffered saline (PBS, Pan Biotech), and cultured for an additional 20 hours in RPMI1640 without _L_-ascorbic acid. Cells were washed two times with PBS, and stained with ref.^29^.

### Heat-map visualization of normalized AUC and hormetic response index

Normalized area under curve (AUC) estimates, measured from the experimental drug response profile and hormetic response index, obtained from statistical consideration of hormetic responses, were used to generate an unbiased clustering of the _L_-ascorbic acid response profile of cancer cells.

### qRT-PCR analysis

Total RNA was extracted from cells using TRI reagent (Molecular Research Center, Cincinnati, OH, USA) according to the manufacturer’s instructions. RNA concentrations were determined by absorbance at 260 nm using a spectrophotometer. cDNA was synthesized using MML-V reverse transcriptase (Bioneer Co, Daejeon, Republic of Korea) according to the manufacturer’s protocols. Quantitative real-time PCR was performed using SYBR Premix Ex Taq (TaKaRa, Otsu, Shinga, Japan) and a Rotor-Gene Q system (Qiagen, Chadstone, Victoria, Australia). Data were analyzed using Rotor-Gene Q series software version 2.3.1 (Qiagen). The following genes were amplified with the indicated primers: p53 (forward 5′-AGGCCTTGGACCTCAAGGATG-3′; reverse 5′-TGAGTCAGGCCCTTCTGTCT-3′), cyclin D1 (forward 5′-GCTGCCAAGTGGAAACCARC-3′; reverse 5′-CCTCCTTCTGCACACATTTGAA-3′), E2F1 (forward 5′-ATGTTTTCCTGTGCCCTGAG-3′; reverse 5′-TGGTGGTGGTGACACTATGG-3′) Ki-67 (forward 5′-ACGCCTGGTTACTATCAAAAGG-3′; reverse 5′-CAGACCCATTTACTTGTGTTGGA-3′).

### Western blotting

Proteins were extracted from cells with a PRO-PREP protein extraction kit according to the manufacturer’s instructions (iNtRON, Sungnam, Korea). Protein concentrations were measured using the Bradford assay (Bio-Rad, Munich, Germany). A total of 30 μg of protein was denatured in sample buffer for 6 minutes at 95 °C. The samples were loaded on 12% SDS-polyacrylamide gels and transferred to nitrocellulose blotting membranes. The membranes were blocked with 5% skim milk in Tris-buffered saline at room temperature for 30 minutes. After three washes in Tris-buffered saline-0.10% Tween 20, the membranes were incubated with anti-Bax (1:2000; 2774; Cell Signaling Technology, Beverly, MA, USA), anti-SVCT2 (1:2500, NBP2-13319, Novus biologics), cyclin D1 (1:2500; NB600-584; Novus biologics), anti-c-Myc (1:2500; NB200-108; Novus biologics), and anti-beta-actin (1:5000; ab20272; Abcam, Cambridge, MA, USA) antibodies at 4 °C overnight. After four washes in Tris-buffered saline-0.10% Tween 20 for 20 minutes, the membranes were incubated with secondary anti-rabbit, anti-rat, or anti-goat antibodies for 1 hour at room temperature. After additional washing, immune-reactive bands were detected using ECL substrate (Pierce, Rockford, IL, USA) and exposure to X-ray film (Agfa-Gevaert N.V., Septestraat, Mortsel, Belgium).

### Detection of ROS generation

Cells were incubated with 20 μM of 2′,7′-Dichlorofluorescin diacetate (Sigma) in culture media for 20 minutes and detached with trypsin and collected in 1 mL of PBS. Cells were washed two times with 500 μL PBS and analyzed on a Guava EasyCyte mini instrument using Cytosoft software version 4.2.1 (Merck Millipore, Billerica, MA, USA).

### _L_-Ascorbic acid uptake

Cells were harvested after 2-hour incubation with 5 mM _L_-ascorbic acid and washed with PBS. The cells were resuspended in 1 mL of PBS with 10% meta-phosphoric acid (MPA) solution and lysed three times by freeze-thaw cycles in a −80 °C deep freezer. The lysate was centrifuged at 16000 rpm at 4 °C for 5 minutes and the supernatant was harvested. Next, 100 μL of sample was mixed with 100 μL of precipitation reagents of vitamin C diagnostics kits (Chromosystems, Gräfelfing, Germany) and incubated for 10 minutes at 4 °C. The mixture was centrifuged at 13000 rpm for 5 minutes and the supernatant was analyzed using a high-performance liquid chromatography (HPLC) system (Shimadzu Corporation, Tokyo, Japan) equipped with Shim-pack CLC-ODS column (6 mm × 15 cm) connected to a Shim-pack G-ODS guard column (4 mm × 1 cm) (Shimadzu). The mobile phase was provided by Chromsystems and the experiment was performed according to the instruction manual. The concentration of _L_-ascorbic acid in cells was determined by manual calculation ${C}_{Analyte,Sample}\,(mg/l)=\frac{{A}_{Sample}\times I{S}_{Standard}}{{A}_{Standard}\times I{S}_{Sample}}\times {C}_{Standard}$. The following instrument settings were used: injection volume 20 μL, run time 10 min, flow rate 1 mL/min, column temperature 25 °C, and UV detector wavelength 245 nm.

## Results

### _L_-Ascorbic acid exhibited anti-cancer effects according to SVCT-2 expression and _L_-ascorbic acid uptake

**Figure 1:**
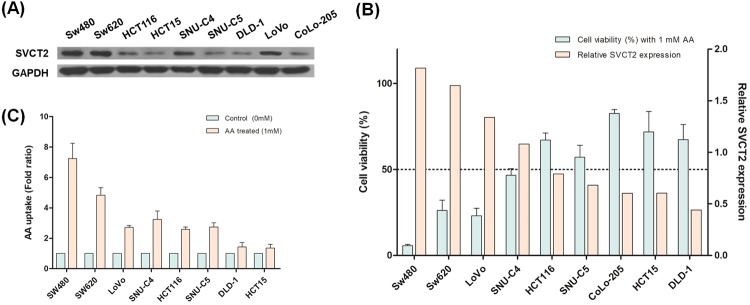
SVCT-2 expression, cytotoxicity, and uptake of _L_-ascorbic acid in colorectal cancer cell lines. (**A**) SVCT-2 expression was analyzed by western blotting. GAPDH was used as a loading control. (**B**) HPLC analysis of the uptake of _L_-ascorbic acid. (**C**) Relative SVCT-2 expression determined with Image J program analysis (black bars) and cell viability with 1 mM _L_-ascorbic acid treatment (white bars).

Previous studies demonstrated that SVCT-2 expression acts as an indicator for high-dose _L_-ascorbic treatment^30^. Therefore, we investigated SVCT-2 expression, _L_-ascorbic acid uptake, and cytotoxic effects of _L_-ascorbic acid in different human colorectal cancer cell lines. The cell lines showed different levels of SVCT-2 expression in western blot analyses **(**Fig. 1A**)**: Sw620, Sw480, and Lovo expressed high levels of SVCT-2 whereas HCT116, HCT15, and DLD-1 expressed low levels. Results of the cell viability assay showed that the cytotoxicity of _L_-ascorbic acid was proportional to SVCT-2 expression **(**Fig. 1B**)**. Moreover, analysis of the uptake of _L_-ascorbic acid by cells using high performance liquid chromatography (HPLC) showed that uptake of _L_-ascorbic acid was also proportional to SVCT-2 expression **(**Fig. 1C**)**.

### Hormetic response to a concentration gradient of _L_-ascorbic acid in low SVCT-2 expressing cell lines

**Figure 2:**
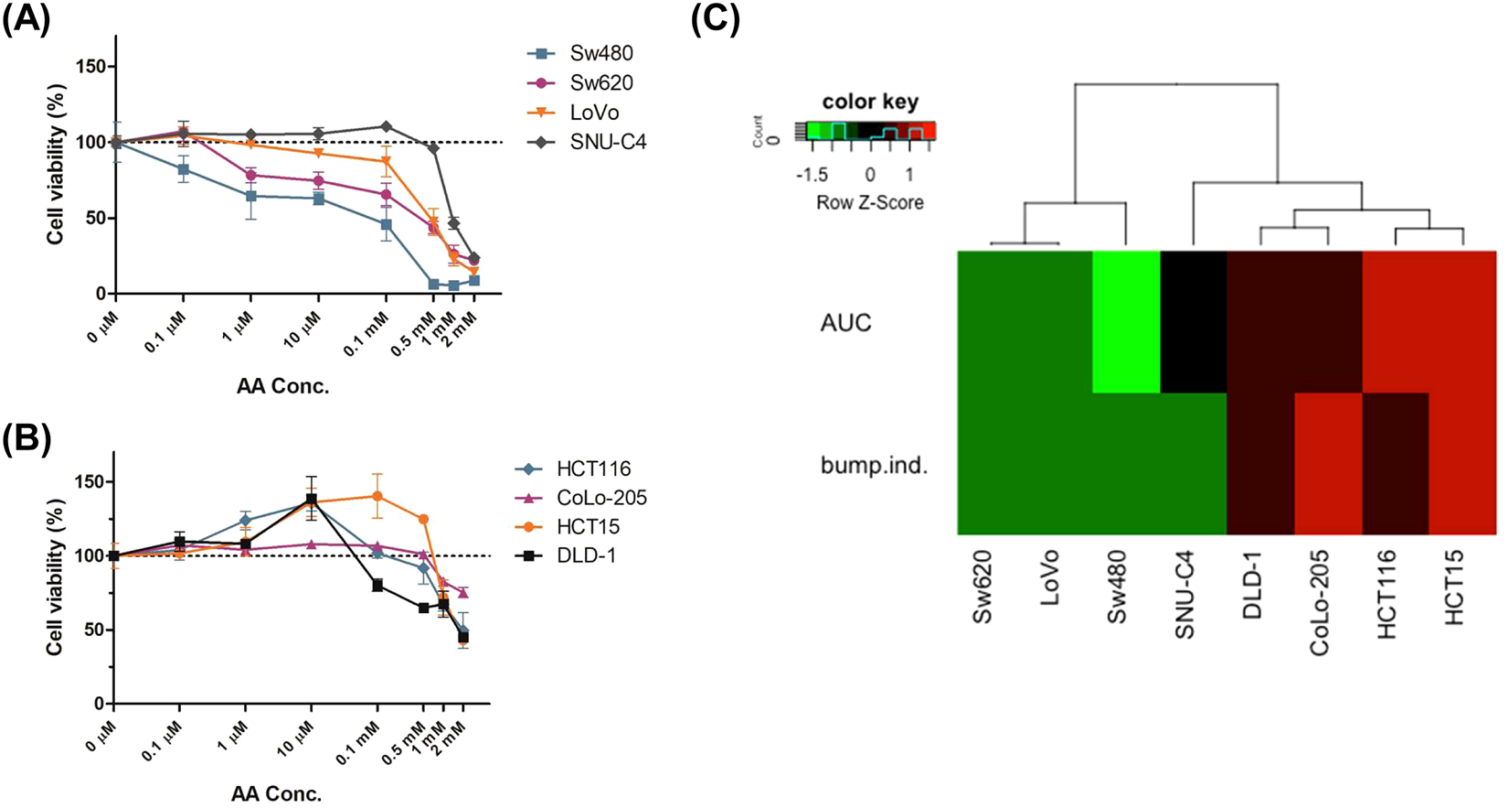
Hormetic response with gradient _L_-ascorbic acid treatment in low SVCT-2 expressing cell lines but not in high SVCT-2 expressing cell lines. (**A**) Cell viability of high SVCT-2 expressing cell lines with gradient _L_-ascorbic acid treatment. (**B**) Cell viability of low SVCT-2 expressing cell lines with gradient _L_-ascorbic acid treatment. (**C**) Clustering and heat-map visualization of the response of eight colorectal cancer cell lines to _L_-ascorbic acid. Drug sensitivity is presented by the area under curve (AUC), and the hormetic responsiveness index, a statistical estimate of the response pattern.

To investigate the cell autonomous impact of _L_-ascorbic acid on cancer cells, the cell viability assay was performed with a gradient of _L_-ascorbic acid concentration. The cancer cell lines displayed differential responses to _L_-ascorbic acid, primarily depending on the expression level of SVCT-2. Some high SVCT-2 expressing cancer cells demonstrated a dramatic cell-autonomous inhibitory effect of _L_-ascorbic acid **(**Fig. 2A**)**. In contrast, low SVCT-2 expressing cell lines showed biphasic responses to _L_-ascorbic acid. This hormetic response to _L_-ascorbic acid in low SVCT-2 expressing cells was characterized by an anti-cancer effect at a high dose (>1 mM) and a pro-proliferative effect at low doses (<10 μM) **(**Fig. 2B**)**. High SVCT-2 expressing cell lines showed anti-cancer effects of _L_-ascorbic acid at all concentrations. These data were further quantitatively assessed with experimental and statistical estimates to generate an unbiased cluster of drug response profile. We particularly focused on the drug sensitivity, presented by the area under curve (AUC), and the hormetic responsiveness index, a statistical estimate of response pattern. Clustering of the data generated three different drug response patterns **(**Fig. 2C**)**. Cluster 1 included Sw620, Sw480, and LoVo and was largely sensitive to _L_-ascorbic treatment. Cluster 2 included SNU C4, which showed an intermediate response to _L_-ascorbic acid. Cluster 3 included DLD-1, Colo-205, HCT116, and HCT15, which were insensitive to _L_-ascorbic treatment and showed a hormetic response. Cluster 3 co-segregated with the low SVCT-2 expressing group.

### ROS generation, apoptotic response, and gene expression analysis in low SVCT-2 expressing cell lines with hormetic response

**Figure 3:**
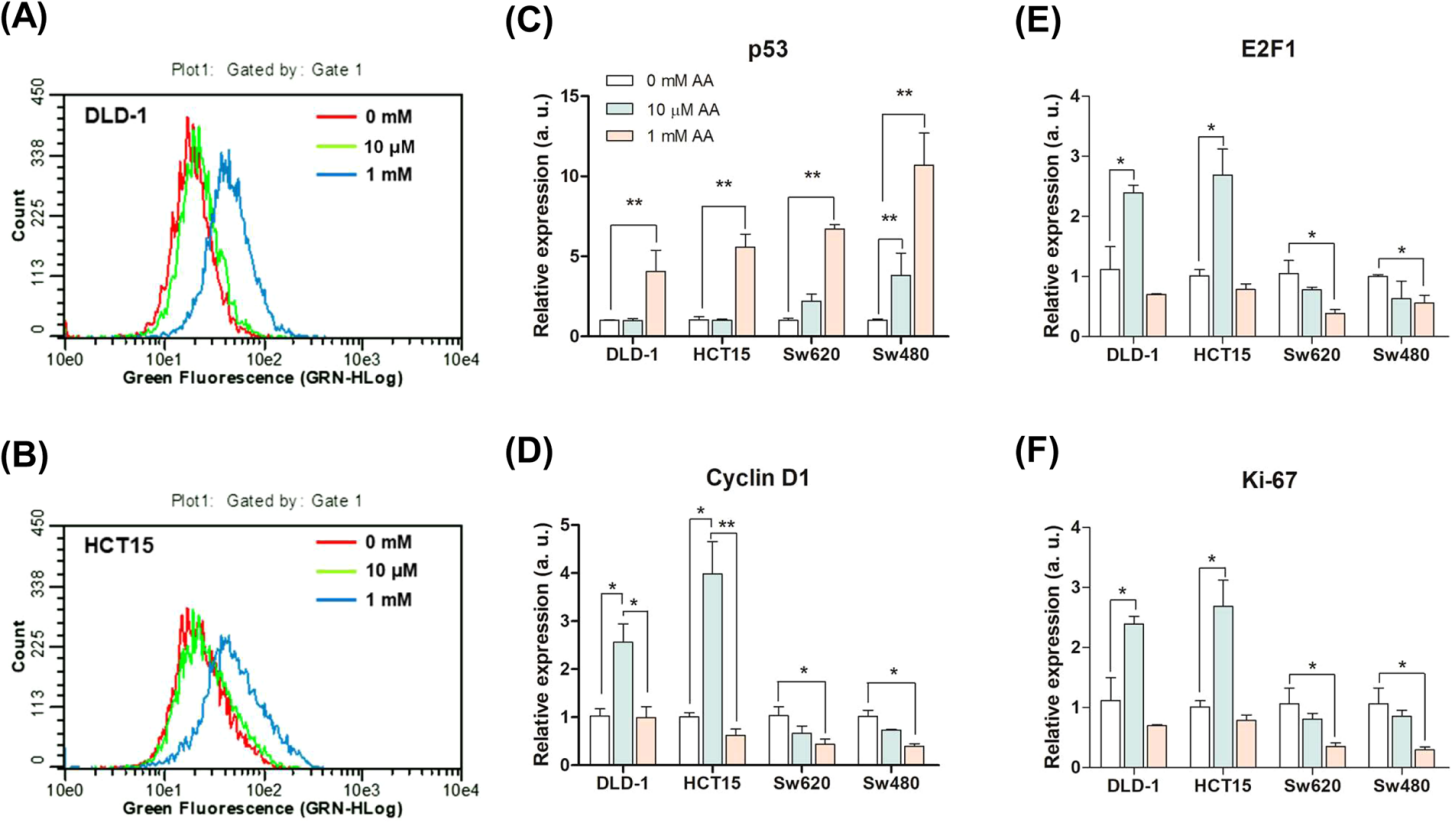
ROS generation in low SVCT-2 expressing cell lines. (**A**) ROS generation was detected by DCF-Da staining in the DLD-1 cell line. (**B**) ROS generation in the HCT15 cell line was detected with 1 mM _L_-ascorbic acid but not with 10 μM _L_-ascorbic acid. (**C**) Expression of p53 in hormetic response condition and apoptotic response condition with 10 μM and 1 mM _L_-ascorbic acid treatment. (**D**–**F**) Expression of cancer cell proliferation markers E2F1, cyclin D1, and Ki-67 in hormetic response condition and apoptotic response condition with 10 μM and 1 mM _L_-ascorbic acid treatment. Data are presented as means ± SEMs. *P < 0.05, **P < 0.005.

Treatment with 1 mM _L_-ascorbic acid induced anti-cancer effects in low SVCT-2 expressing cell lines whereas 10 μM _L_-ascorbic acid promoted proliferation of these cells. To investigate ROS generation induced by 1 mM and 10 μM _L_-ascorbic acid, low SVCT-2 expressing cell lines were stained with 2′,7′-Dichlorofluorescin diacetate (DCF-Da) after _L_-ascorbic acid treatment **(**Fig. 3A,B**)**. In the low SVCT-2 expressing cell lines DLD-1 and HCT15, ROS were generated with 1 mM _L_-ascorbic acid treatment but not with 10 μM _L_-ascorbic acid. To confirm apoptotic and hormetic proliferation responses with _L_-ascorbic acid treatment, a quantitative real-time polymerase chain reaction (qRT-PCR) was performed for gene expression analysis. Expression of tumor protein 53 (p53) indicated apoptotic responses after treatment with 10 μM and 1 mM _L_-ascorbic acid in high SVCT-2 expressing cell lines; however, in low SVCT-2 expressing cell lines, only 1 mM _L_-ascorbic acid treatment induced p53 expression **(**Fig. 3C**)**. Cyclin D1 expression was analyzed for determination of proliferation **(**Fig. 3D**)**. In addition, expression of E2F transcription factor 1 (E2F1) and Antigen KI-67 (Ki-67) was increased in low SVCT-2 expressing cell lines after treatment with 10 μM _L_-ascorbic acid treatment **(**Fig. 3E,F**)**. Low-dose _L_-ascorbic acid treatment induced a hormetic dose response in low SVCT-2 expressing cell lines DLD-1 and HCT15 and also induced cyclin D1, E2F1, and Ki-67 expression. However, in high SVCT-2 expressing cell lines Sw620 and Sw480, the expression of cyclin D1 decreased after treatment with both 10 μM and 1 mM _L_-ascorbic acid. These results suggest that _L_-ascorbic acid induced anti-cancer effects at both high and low doses in high SVCT-2 expressing cell lines. In contrast, in low SVCT-2 expressing cell lines, high-dose _L_-ascorbic acid exhibited anti-cancer effects as evidenced by expression of p53, whereas low-dose _L_-ascorbic acid treatment induced genes associated with cell proliferation.

### Molecular analysis of hormetic response of _L_-ascorbic acid treatment in low SVCT-2 expressing cell lines

**Figure 4:**
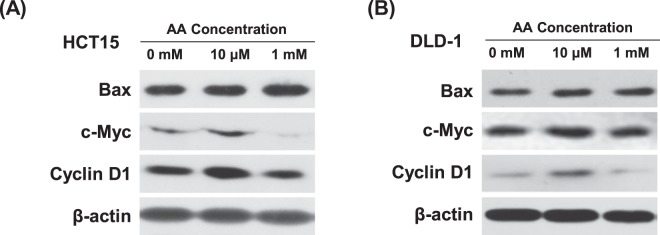
Expression of cancer proliferation markers in low SVCT-2 expressing cell lines. (**A**,**B**) Expression of c-Myc and cyclin D1 was analyzed by western blotting in HCT15 and DLD-1 cell lines after _L_-ascorbic acid treatment. β-actin was used as a loading control.

To further investigate apoptosis and proliferation of cancer cells in response to _L_-ascorbic acid treatment at the protein expression level, western blot analysis was performed after treatment with low and high concentrations of _L_-ascorbic acid. Low SVCT-2 expressing cell lines HCT15 and DLD-1 showed increased c-Myc and cyclin D1 expression as a proliferative response after treatment with 10 μM _L_-ascorbic acid but decreased expression of these proteins with 1 mM _L_-ascorbic acid **(**Fig. 4A,B**)**. After treatment with 10 μM _L_-ascorbic acid, expression of cyclin D1 was increased and co-localization of cyclin D1 with CDK4 was observed by confocal microscopy (Supplementary Figs 2 and 3).

### Hormetic response in high SVCT-2 expressing cell lines with SVCT-2 family inhibitors

**Figure 5:**
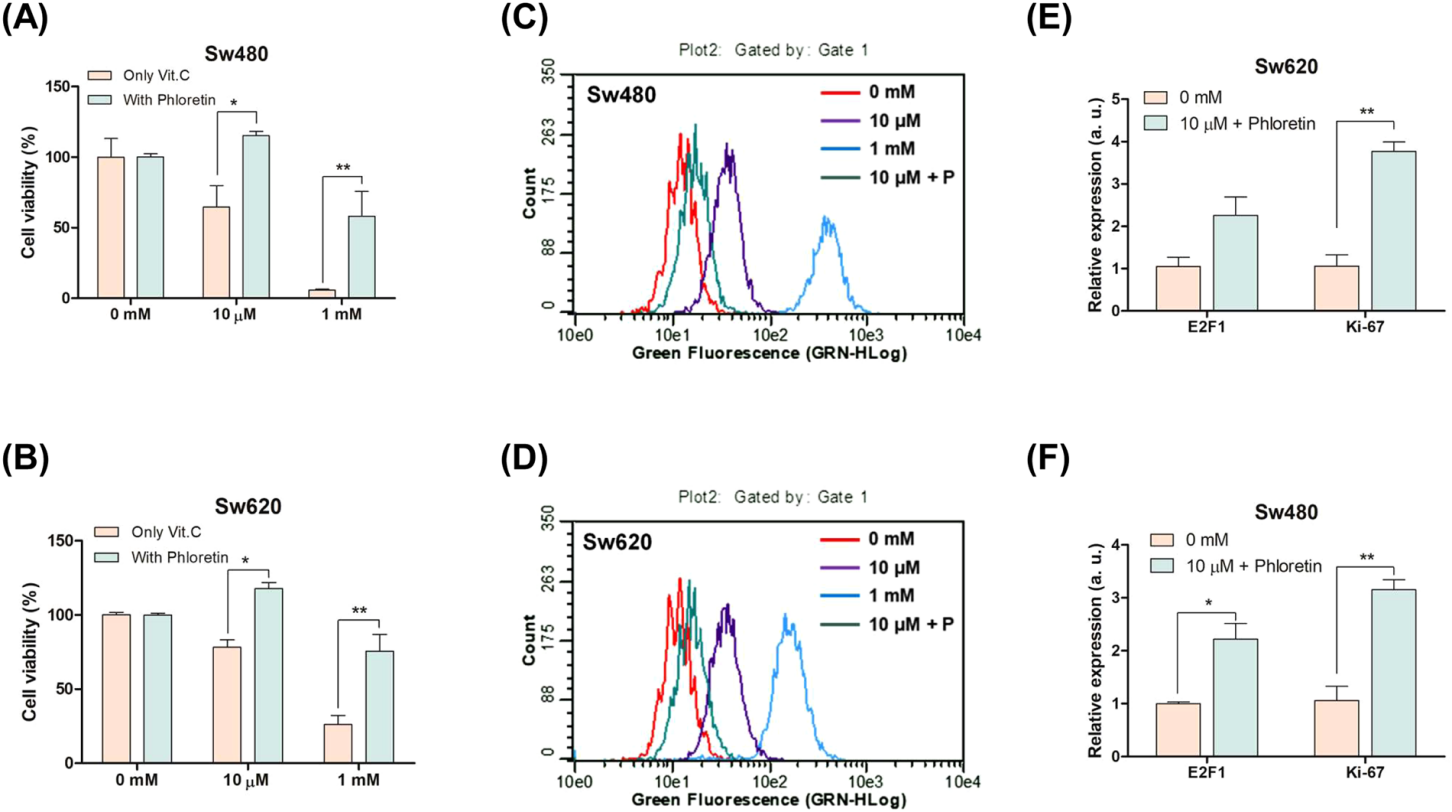
Hormetic response in high SVCT-2 expressing cell lines with SVCT-2 inhibition. (**A**,**B**) Cell viability assay in Sw620 and Sw480 cell lines treated with 10 μM or 1 mM _L_-ascorbic acid and phloretin. (**C**,**D**) ROS assay by DCF-Da staining in Sw620 and Sw480 cell lines treated with 10 μM or 1 mM _L_-ascorbic acid and phloretin. Data are presented as means ± SEMs. *P < 0.05, **P < 0.005.

To determine whether the hormetic proliferation response was mediated through _L_-ascorbic acid uptake via SVCT-2, we treated high SVCT-2 expressing cell lines with 1 mM or 10 μM _L_-ascorbic acid in combination with phloretin as a SVCT family inhibitor. The concentration of phloretin was confirmed to be non-toxic by a cell viability assay (Supplementary Fig. 1A). _L_-Ascorbic acid uptake in Sw480 and Sw620 cell lines was inhibited by phloretin in the presence of 1 mM _L_-ascorbic acid (Supplementary Fig. 1B). With inhibition of the SVCT family, high SVCT-2 expressing cell lines showed a hormetic proliferation response when treated with 10 μM _L_-ascorbic acid **(**Fig. 5A,B**)**. These results suggest that the hormetic proliferation response can be triggered when an insufficient amount of _L_-ascorbic acid is delivered to cancer cells.

### ROS generation in apoptotic response and hormetic response of high SVCT-2 expressing cell lines

In high SVCT-2 expressing cell lines Sw620 and Sw480 both 1 mM and 10 μM _L_-ascorbic acid induced an apoptotic response, but upon inhibition of SVCT-2 these cells showed a hormetic proliferation response. Since inhibition of SVCT with phloretin induced hormetic proliferation with 10 μM _L_-ascorbic acid, we investigated ROS generation via DCF-Da staining. ROS generation was induced by treatment with 1 mM and 10 μM _L_-ascorbic acid. However, inhibition of SVCT-2 by phloretin decreased ROS generation **(**Fig. 5C,D**)**.

### Expression of cancer cell proliferation markers in high SVCT-2 expressing cell lines

**Figure 6:**
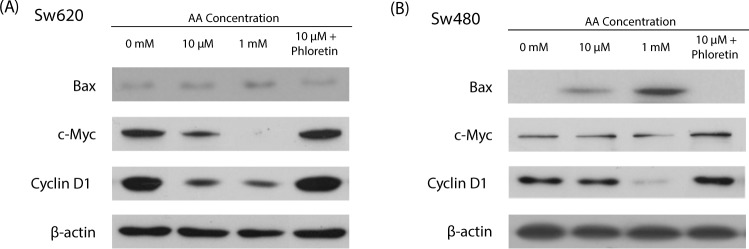
Expression of cancer proliferation markers and in high SVCT-2 expression cell lines. (**A**,**B**) Western blot analysis of c-Myc and cyclin D1 in Sw620 and Sw480 cell lines after co-treatment with _L_-ascorbic acid and phloretin. β-actin was used as a loading control.

To analyze the hormetic proliferation response induced by inhibition of SVCT-2 with phloretin, the expression of cancer proliferation markers and BAX was analyzed by qRT-PCR and western blotting. Expression of Ki-67 and E2F1, which were used as cancer proliferation markers, was investigated by qRT-PCR. The results showed increased expression of Ki-67 and E2F1 in Sw620 and Sw480 cell lines treated with 10 μM _L_-ascorbic acid and phloretin **(**Fig. 6A,B**)**. Also, in western blot analysis, c-Myc and cyclin D1 protein expression was significantly decreased by treatment with 1 mM and 10 μM _L_-ascorbic acid but increased under hormetic proliferation conditions in the presence of phloretin **(**Fig. 6C,D**)**.

## Discussion

The controversy surrounding _L_-ascorbic acid cancer therapy began with conflicting clinical results from Linus Pauling’s study and the Mayo Clinic study^12–15^. To resolve this controversy, numerous studies have focused on the anti-cancer effect of _L_-ascorbic acid and its mechanism^1–4,8,10^. Some studies demonstrated that cytotoxic action of _L_-ascorbic acid mediated by oxidative DNA breakage in lymphocyte considered with cellular copper ion levels^5,6,31^. Hadi *et al*. (2011) demonstrated importance of chromatin-bound copper in the pro-oxidant cellular DNA breakage by ascorbic acid. Similar with other plant derived pro-oxidants such as thymoquinone, epicatechin and eoigallocatechin-3-gallate, _L_-ascorbic acid generated ROS in cancer cells via SVCT2 and led to cell death^32–34^. Other studies revealed that SVCT-2 expression is a determination factor of _L-_ascorbic acid cancer treatment^30^ and that hypoxia-inducible factor–positive cells are susceptible to _L_-ascorbic acid treatment^16^. Other studies developed cancer therapy regimens that combined _L_-ascorbic acid with chemotherapeutic agents or other agents^35–37^. Although these research studies revealed the action mechanism and developed more effective application methods for _L_-ascorbic acid cancer therapy there was no explanation for why the group of patients who were treated with _L_-ascorbic acid in the Mayo Clinic study showed a lower survival rate than the placebo group^14,15^. To interpret these results we investigated the action of _L_-ascorbic acid on cancer cells using a gradient concentration of 1 μM to 2 mM based on pharmacokinetics results for _L_-ascorbic acid^17^.

**Figure 7:**
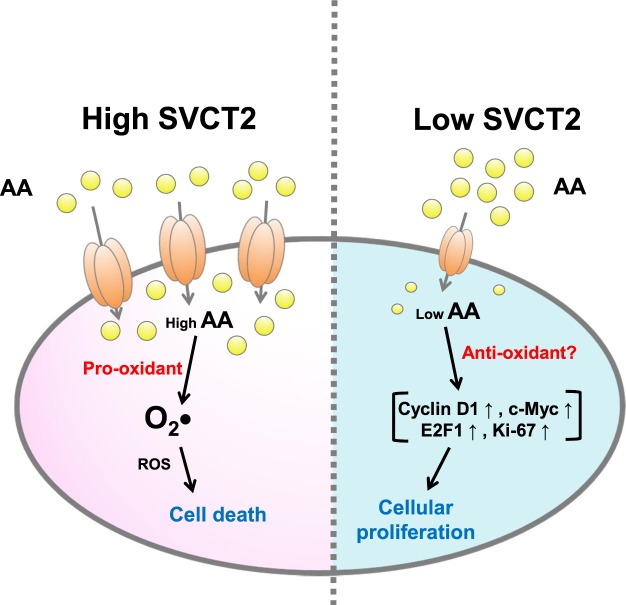
Proposed biphasic role of _L_-ascorbic acid in cancer cells according to SVCT-2 expression.

It was previously reported that SVCT-2 expression is an indicator for _L_-ascorbic acid treatment^30^. In this report we confirmed that SVCT-2 functions as a _L_-ascorbic acid transporter and that the anti-cancer effects of _L_-ascorbic acid are proportional to SVCT-2 expression of cell lines. To interpret results from the Mayo Clinic and other past clinical studies^12–15^ we proposed that _L_-ascorbic acid has a biphasic role in cancer cells depending on _L_-ascorbic acid uptake, which is in turn dependent on SVCT-2 expression levels. Our results demonstrated that if there was a sufficient concentration of _L_-ascorbic acid to generate ROS it functions as an anti-cancer agent regardless of SVCT-2 expression, whereas a hormetic proliferation response was observed with low SVCT-2 expressing cell lines in which there was insufficient uptake of _L_-ascorbic acid to generate ROS. In high SVCT-2 expressing cell lines, _L_-ascorbic acid showed cell growth inhibition and apoptotic responses at all concentrations of _L_-ascorbic acid. Therefore, with sufficient delivery into cancer cells via a high SVCT2 expression level _L_-ascorbic acid is an effective chemotherapeutic agent but deficient delivery of _L_-ascorbic acid to cancer cells due to low SVCT2 instead increases the proliferation activity of cancer. Cyclin D1 has prognostic significance through its function in cancer cell proliferation^38–40^, and also plays an essential role in recruiting transcription factors such as E2F1 and regulates gene transcription via inhibition of p300^41,42^. According to our results, cyclin D1, which was induced by a low concentration of _L_-ascorbic acid, is a significant factor in the hormetic proliferation response. It remains to be elucidated which proteins or signaling molecules are stimulated by ascorbic acid, but our results suggest that cyclin D1 and cyclin-dependent kinase (CDK4) co-localization could be a critical factor for cell proliferation (Supplementary Figs 2 and 3)^43^. In addition, increased expression of c-Myc, Ki-67, and E2F1, cyclin D1–related proliferation markers, further supports results of the cell viability assay. Ki-67 and c-Myc increase cell proliferative activity through DNA binding^40,44,45^. These results are summarized in Fig. 7.

Our findings indicate that _L_-ascorbic acid has effective chemotherapeutic potential in high SVCT-2 expressing cancer cells. However, an insufficient dose of _L_-ascorbic acid stimulates cancer cell proliferation, which is mediated by cyclin D1. Therefore, high-dose _L_-ascorbic acid cancer therapy is an effective treatment for high SVCT-2 expressing cancer with few side effects to patients. However, for low SVCT-2 expressing cancers _L_-ascorbic acid might not only be less effective, but could also be deleterious to cancer patients. This hypothesis may explain the conflicting clinical results from two different research groups in which the SVCT-2 expression status of patients was unknown^12–15^.

In addition, we expect that our results are followed from anti-oxidant property of _L_-ascorbic acid. Some studies shown anti-oxidants expediting cancer progression^46,47^. Sayin *et al*. (2014) showed that N-acetylcysteine (NAC) and vitamin E reduced ROS in cancer cell and increased proliferation of cancer cell. These results demonstrate that when oxidative stress reduced, cancer cells are more proliferative because endogenous anti-oxidant related oncogene nuclear factor erythroid-2–related factor 2 reduced ROS thereby increased cancer proliferation^48–50^. It is possible that other anti-oxidant such as NAC, Vitamin E, glutamine and others could show similar hormetic proliferation response like _L_-ascorbic acid.

We demonstrated that high-dose _L_-ascorbic acid cancer therapy requires careful consideration with regard to generating a sufficient concentration of _L_-ascorbic acid inside cancer cells. Also, determination factors for _L_-ascorbic acid cancer therapy in addition to SVCT-2 and hypoxia-inducible factor^16,30^ should be discovered for more effective cancer therapy. Our findings also suggest that the combination of high-dose _L_-ascorbic acid cancer therapy with SVCT-2 inducible agents or synergistic chemotherapeutic agents could be a more effective cancer therapy and may resolve the current controversies surrounding _L_-ascorbic acid cancer therapy.

## Electronic supplementary material

Supplementary data
