# Supplementary material for: Hormetic dose response to L-ascorbic acid as an anti-cancer drug in colorectal cancer cell lines according to SVCT-2 expression
Source: Sci Rep. 2018 Jul 27;8:11372. doi: 10.1038/s41598-018-29386-7 (PMC6063950; doi:10.1038/s41598-018-29386-7)
Supplement: Supplementary file 1 — Supplementary data [file 41598_2018_29386_MOESM1_ESM.docx]

**Hormetic dose response to _L_-ascorbic acid as an anti-cancer drug in colorectal cancer cell lines according to SVCT-2 expression**

Sungrae Cho^1^, Jin Sung Chae^2^, Hocheol Shin^1^, Yujeong Shin^3^, Haeun Song^3^, Youngwook Kim^4^, Byong Chul Yoo^5^, Kangsan Roh^1^, Seungchan Cho^1^, Eui-joon Kil^1^, Hee-seong Byun^1^, Sang-ho Cho^1^, Seyeon Park^3^, Sukchan Lee^1*^ and Chang-Hwan Yeom^2*^

^1^Department of Genetic Engineering, Sungkyunkwan University, Suwon, 16419, Republic of Korea

^2^Yeom Chang-Hwan hospital, Seoul, 06605, Republic of Korea

^3^Department of Applied Chemistry, Dongduk Women’s University, Seoul, 02748, Republic of Korea

^4^Department of Health Sciences and Technology, Samsung Advanced Institute for Health Sciences and Technology, Sungkyunkwan University, Seoul, 06351, Republic of Korea

^5^Colorectal Cancer Branch, Division of Translational and Clinical Research, Research Institute, National Cancer Center, Goyang, 10408, Republic of Korea

*Corresponding author: Chang-Hwan Yeom, Sukchan Lee*

Chang-Hwan Yeom

Yeom Chang-Hwan hospital, Seoul, 06605, Republic of Korea

Tel: +82-2-514-1249; e-mail: [lymphych@hanmail.net](mailto:lymphych@hanmail.net)

Sukchan Lee

Dept. of Genetic Engineering, Sungkyunkwan University, Suwon, 16419, Korea

Tel: +82-31-290-7866; FAX: +82-31-290-7870; e-mail: [cell4u@skku.edu](mailto:cell4u@skku.edu)


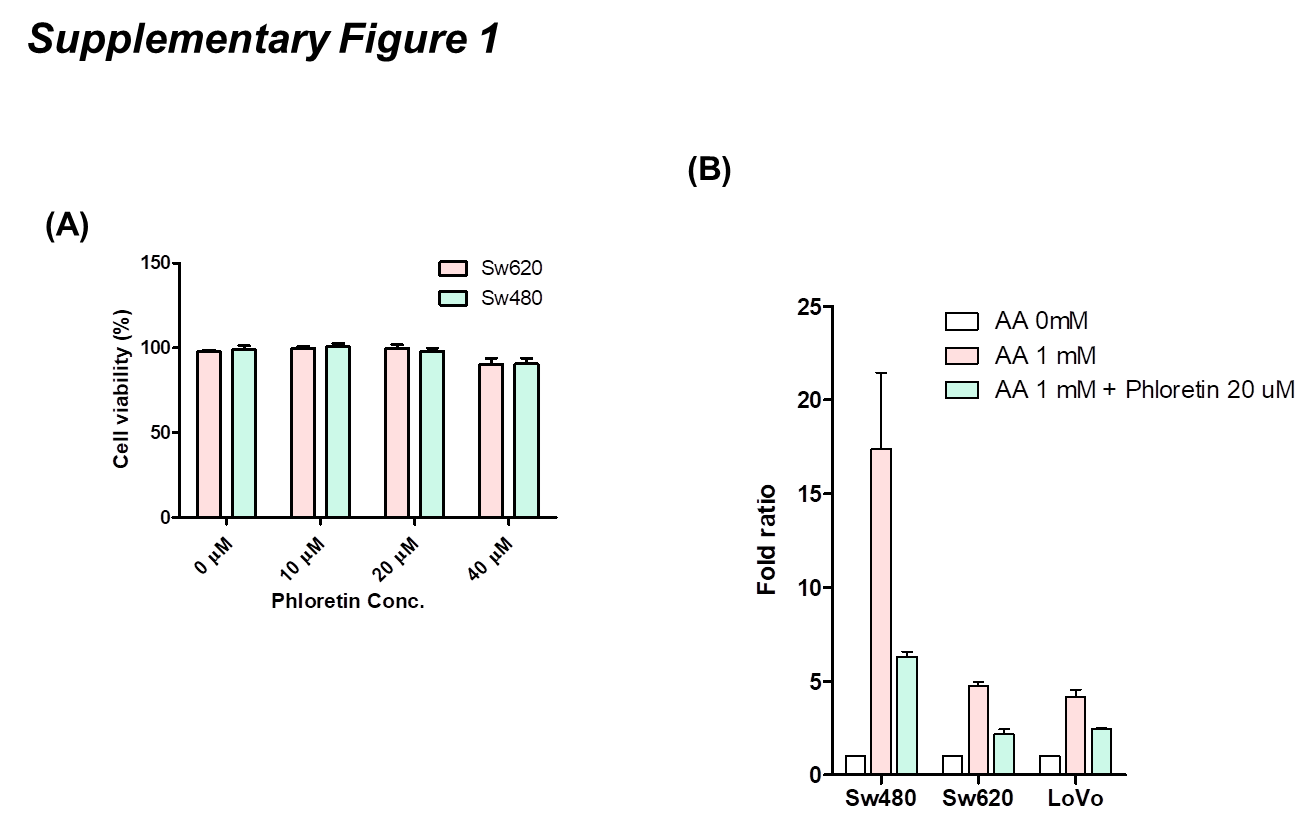


**Supplementary Figure 1. A.** Cell viability assay for phloretin in Sw620 and Sw480 cell lines. Cell cytotoxicity was not observed with 20 μM phloretin treatment in both cell lines. **B.** _L_-Ascorbic acid uptake in cancer cells was analyzed by HPLC. Treatment with phloretin inhibited _L_-ascorbic acid uptake.


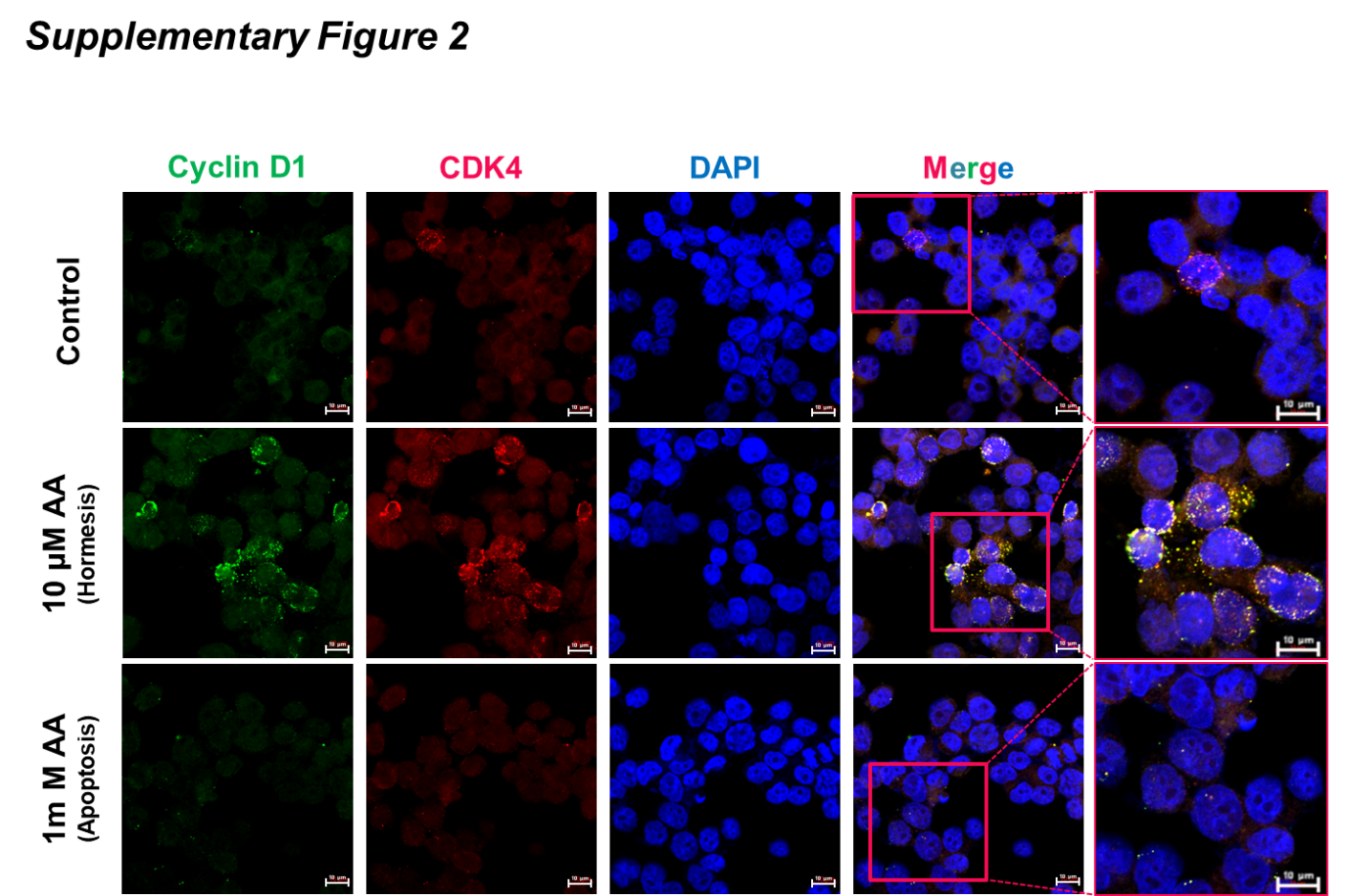


**Supplementary Figure 2.** Co-staining of cyclin D1 and CDK4 by immunocytochemistry in DLD-1 cell line. Cyclin D1 and CDK4 were co-localized in nucleus and cytosol after treatment with 10 μM _L_-ascorbic acid.


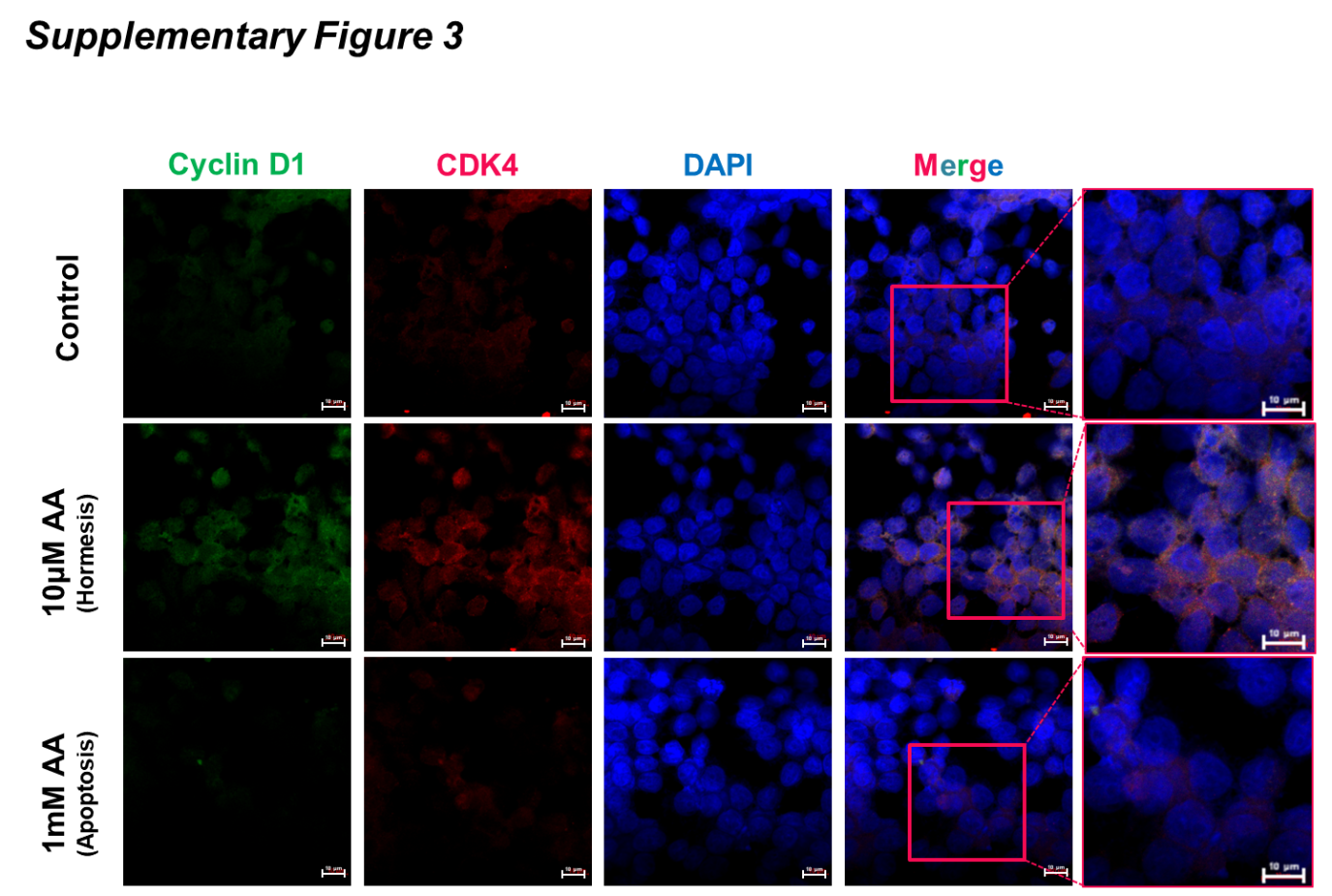


**Supplementary Figure 3.** Cyclin D1 and CDK4 localization was analyzed in HCT15 cells by confocal microscopy. Co-localization of Cyclin D1 and CDK4 was observed in nuclei and cytosol after treatment with 10 μM _L_-ascorbic acid.


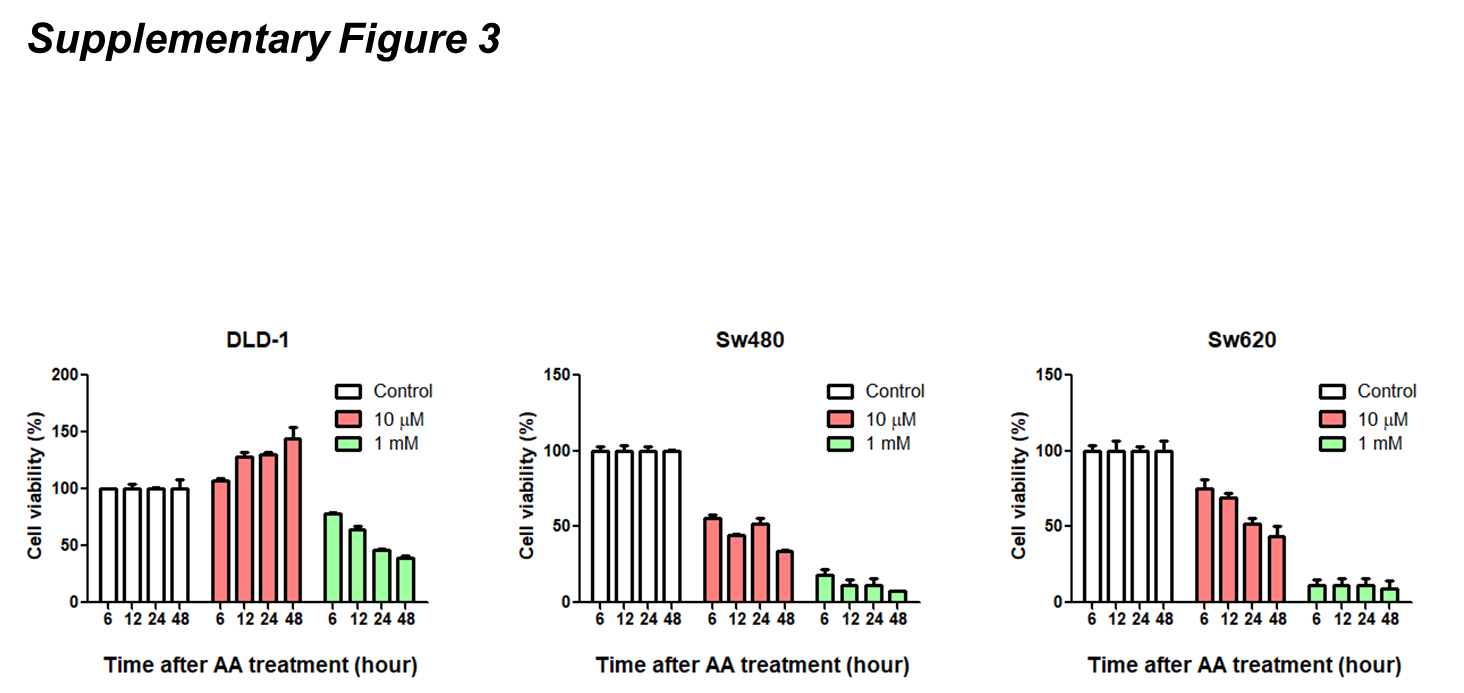


**Supplementary Figure 4.** Time-course cell viability assay was analyzed in DLD-1, Sw620 and Sw480 cell line. 10 μM and 1mM _L_-ascorbic acid was treated. Hormetic proliferation response was observed after 12 hr with 10 μM of _L_-ascorbic acid treatment.
